# Alcohol-Induced Deaths Among American Indian and Alaska Native Individuals—“Drinking Was What I and Others Just Did”

**DOI:** 10.1001/jamanetworkopen.2019.21391

**Published:** 2020-02-05

**Authors:** Spero M. Manson

**Affiliations:** Centers for American Indian and Alaska Native Health, Colorado School of Public Health, University of Colorado, Aurora

“Alcohol was a way of life for me, my family, my friends, my community. I used to think being Indian was about drinking. That I couldn’t stop because I was Indian… and if I did, I would no longer be Indian. The bad things about being a drunk were all around me—liver disease, domestic violence, car accidents, suicide, PTSD [posttraumatic stress disorder]. But I never really thought about dying. Drinking was what I and others just did.”

The article by Spillane et al^1^ awakened my memory of this reflection, shared by an Alaska Native patient in a tribal residential treatment program with which I work. His words acknowledged the widespread consumption of alcohol in many of these communities, its interface with personal identity, the acceptance of alcoholism as an inevitable part of life, and the denial of its harmful consequences, apparent as they may be. Tribes have made remarkable progress in reducing the stigma surrounding addiction in their communities and in developing prevention as well as treatment programs. There are bright lights of success in curbing the nature, extent, and consequences of alcohol use and dependence. Yet, as the trends described by Spillane et al^1^ underscore, alcohol-induced mortality in general continues to rise and heralds many battles still to be fought in Native communities.

A major strength of the article by Spillane et al^1^ is the location of these trends within the broader national landscape, not just in comparison with white individuals as the primary reference group, which is typically the case. In this regard, Spillane et al^1^ report that in 2016, the age-standardized rates of alcohol-induced deaths were highest among American Indian and Alaska Native (AIAN) men and women, substantially greater than those among their counterparts in the other major racial/ethnic populations. Moreover, these rates increased steadily and significantly for both sexes during the 17-year period of observation. This increase among AIAN individuals stands in sharp contrast to a significant decrease among black individuals, little or no change among Latino individuals, and a general but less significant increase among white individuals. Consistent with the early onset and more prodigious consumption of alcohol among AIAN individuals, their peak mortality occurred earlier (ie, ages 45–59 years) compared with all other groups (ie, ages 55–64 years). Lastly, the rates of alcohol-induced deaths tended in general to be highest in the western United States; most AIAN individuals live west of the Mississippi River. By placing these observations among the population at large as well as other subgroups at risk, the authors depart from the all too common practice of singling out AIAN individuals and, thereby, reduce the likelihood of further stigmatizing the latter’s particular struggle with alcohol use and dependence. The findings justify the authors’ call to arms to address alcohol-related morbidity and mortality of this long-recognized yet growing public health crisis among Native people and other segments of the US population.

Spillane et al^1^ appropriately acknowledge several limitations of their work and the ensuing results. Those not mentioned are worth further consideration in this instance and by others as they pursue similar lines of inquiry. Early in the article, the authors underscore the challenge of accounting for racial misclassification in the death certificates used to enumerate alcohol-induced deaths. However, they fail to mention the implications of this very likely and potentially substantial shortcoming for the findings and the confidence readers can place in them. Espey et al^2^—and many other investigators—emphasize this persistent problem, which bears repeating. Because the usual implication of racial misclassification in the case of AIAN individuals is to underestimate the condition of interest, the high reported rates of alcohol-induced deaths render the observed rates even more concerning.

It also is important to acknowledge that more than 72% of AIAN individuals live in urban and suburban areas^3^; the article by Spillane et al^1^ focuses largely on rural, reservation-dwelling Native people. Caution is in order about generalizing these findings to all AIAN individuals, when, in fact, they do not speak to nearly three-quarters of this population. A related concern revolves around the manner in which we estimate trends for small populations and then extrapolate those trends to national levels for comparison with other, larger subgroups.^4^

With respect to rurality, Spillane et al^1^ operationalize residence in terms of Purchased Referred Care, a common convention that other studies characterize as Contract Health Service Delivery Areas or Tribal Service Delivery Area counties. Careful attention to the strengths and weakness of these decisions is in order. Regional variation is extremely important, given that social determinants of health differ geographically in terms of poverty level, health care access, and other factors.^5^ For example, the highest death rates for a wide range of health conditions are evident in Alaskan and Northern and Southern Plains Contract Health Service Delivery Area counties, which reflect such differences.^6^ Following the implementation of the Patient Protection and Affordable Care Act, Purchased Referred Care payments have varied by state Medicaid expansion status. Consequently, many low-income AIAN individuals qualify for Medicaid and do not need Purchased Referred Care to cover costs, which may affect assumptions about the role that residence in these areas plays in alcohol-induced deaths.

Apart from the epidemiology of alcohol-induced mortality and associated risks, what other circumstances might contribute to our understanding of these trends? Studies of alcohol detoxification programs in AIAN communities shed light on this question. Detoxification is a critical first step in preparing individuals to treat their alcohol use and dependence. To my knowledge, the only study to date of this transition among Native peoples revealed that 78% of AIAN participants in an Alaska-based program completed detoxification, which fell within the reported range of other studies for the general population (ie, 53%–88%).^7^ However, of those who completed detoxification, 36% subsequently accepted referral to and 21% actually entered into alcohol use and dependence treatment programs^7^; these rates are substantially lower than those among other populations. Ongoing work among another detoxification program in a different region of Alaska revealed that, during a 5-year period, less than 1% of more than 1100 unique, largely AIAN participants in a medical detoxification program successfully transitioned to alcohol use and dependence treatment within 1 year of discharge (S.M.M., unpublished data, 2019). These findings imply a revolving door of admissions to detoxification that accomplishes little with respect to addressing the needs of those who experience the consequences of alcohol use and dependence, notably the increased morbidities that heighten their risk of death. The availability of all the treatment programs in the world holds little hope of making a difference if we cannot successfully support the transition to appropriate care for those most in need. This challenge precedes others, including whether the options most likely adopted by AIAN communities offer the best, most well-informed, and most effective treatments.^8^

As Spillane et al^1^ note, alcohol-induced deaths are the tip of the iceberg. Alcohol-related deaths—notably suicide, motor vehicle collisions, drowning, and homicide—remain high among AIAN individuals and continue to rise. Combined, they underscore the urgency of the authors’ call to action. Recent concerns about the opioid epidemic sweeping through AIAN populations are well founded and demand immediate attention. However, attendant shifts in funding and programming emphases threaten to overshadow the continued, growing crisis of alcohol use and dependence in this population. We forget this peril at great risk to the future of Native peoples.
